# A strong wink between verbal and emoji-based irony: How the brain processes ironic emojis during language comprehension

**DOI:** 10.1371/journal.pone.0201727

**Published:** 2018-08-15

**Authors:** Benjamin Weissman, Darren Tanner

**Affiliations:** Department of Linguistics, University of Illinois, Urbana, Illinois, United States of America; Icahn School of Medicine at Mount Sinai, UNITED STATES

## Abstract

Emojis are ideograms that are becoming ubiquitous in digital communication. However, no research has yet investigated how humans process semantic and pragmatic content of emojis in real time. We investigated neural responses to irony-producing emojis, the question being whether emoji-generated irony is processed similarly to word-generated irony. Previous ERP studies have routinely found P600 effects to verbal irony. Our research sought to identify whether the same neural responses could also be elicited by emoji-induced irony. In three experiments, participants read sentences that ended in either a congruent, incongruent, or ironic (wink) emoji. Results across all three experiments demonstrated clear P600 effects, the amplitudes of which were correlated with participants’ tendency to treat the emoji as a marker of irony, as indicated by behavioral comprehension question responses. These ironic wink emojis also elicited a strong P200 effect, also found in studies of verbal irony processing. Moreover, unexpected emojis (both mismatch and ironic emoji) also elicited late frontal positivities, which have been implicated processing unpredicted words in context. These results are the first to identify how linguistically-relevant ideograms are processed in real-time at the neural level, and specifically draw parallels between the processing of word- and emoji-induced irony.

## Introduction

Emojis are graphical symbols easily inserted into Computer Mediated Communication that have recently exploded in use and popularity. Articles about emojis have been published in venues like *The New York Times*, *The New Yorker*, and *Time* magazine [1, 2, 3], and *The Emoji Movie* debuted in theaters in July 2017, albeit to horrible reviews. Popular media aside, a few scholarly investigations into emojis have shed light on their communicative properties and tendencies, including the ability to fulfill a wide range of linguistic functions. Broadly speaking, the use of emojis in conjunction with “traditional” written language can be seen as a type of multimodal interaction that combines more than one modality systematically into a single communicative utterance (e.g., [4, 5, 6, 7]).

Emoticons, the predecessor to newer emojis, were originally regarded as simple markers of emotions (e.g., [8, 9]) but more recent approaches (e.g., [10, 11]) have posited that they can serve many complex pragmatic functions, a claim that is further bolstered by the recent explosion in number and usage frequency of emojis. Some recent research has investigated the ambiguity and potential for miscommunication that emojis carry, even when embedded in linguistic contexts [12, 13, 14].

One conventional–and less ambiguous–usage of a specific emoticon/emoji is the wink-face symbol used to indicate sarcasm or irony; though not the only usage of this symbol, the sarcasm/irony usage has been demonstrated with consistency in both intent and uptake (e.g., [15, 16, 17]). This usage is consistent with Dresner & Herring’s (2010) theory claiming that emoticons can be used to indicate illocutionary force–the speaker’s intended purpose in performing a given utterance [18, 19]. Nonetheless, despite the fact that emoji use is common, even ubiquitous, in current electronic communication, no work has yet investigated how emojis and their communicative functions are processed in language contexts in real time. Here we focus specifically on the processing of emojis used to signal irony/sarcasm and ask 1) how emoji-related irony is processed at the neural level using event-related brain potentials (ERPs), and 2) whether emoji-induced irony elicits brain responses that are qualitatively similar to word-induced irony seen in prior work.

Several studies have used ERPs to study word-induced irony, typically by time-locking brain responses to discourse-final words that were either consistent with the prior discourse context or were unexpected in an ironic way [20, 21, 22, 23]. Across studies, results have been consistent, with ironic completions eliciting a biphasic response characterized by an enhanced P200 component followed by a P600. The P200 is typically interpreted as reflecting attention-related processes (e.g., [24, 25]) while the P600 has traditionally been associated with syntactic processing or syntactic integration (e.g., [26, 27]). However, more recent work has shown that the P600 is not a reflection of syntax specifically, but general integration difficulty, enhanced monitoring processes, or general reanalysis (e.g., [28, 29, 30]). Overall, this prior work has argued that irony is not immediately and directly accessed, as has been claimed by some (e.g., [31, 32, 33]), but instead provides support for theories claiming multi-stage processing of irony (e.g., [34, 35, 36]), with the reanalysis P600 taken as a marker of an increased and delayed processing load.

The P600 elicited in these studies contrasts with another well-studied component associated with language comprehension, the N400. The N400 component is elicited by all meaningful stimuli, but its amplitude in language studies is well known to be modulated by a number of factors including word predictability, length, frequency, and semantic fit with a prior sentence or discourse context [37]. Extending this line of research to multimodal communication involving emojis, one might expect that, to the extent that emojis are meaningful stimuli, emojis conveying an unexpected meaning (e.g., mismatching in valence with a prior context) might elicit a larger N400 amplitudes than expected emojis. Additionally, a number of ERP studies have also reported a late frontal positivity (henceforth LFP) in response to unexpected words, which were unpredictable based on prior context [38, 39, 40, 41]. An additional prediction is that unexpected emojis might elicit this late frontal positivity in addition to or perhaps instead of the more posteriorly distributed P600 and centrally distributed N400.

With theoretical work on the linguistic and pragmatic functions of emojis suggesting the symbols can have similar functions as words in indicating irony (e.g., [10, 15]), it remains unknown how emojis are processed in meaningful contexts in real time, and whether emojis signaling irony elicit neural responses similar to word-induced irony (that is, P200 + P600). Such an isomorphism would be consistent with the hypothesis that a common set of processes underlies the processing of irony signaled both traditionally and ideographically. Additionally, our experimental design allows for the study of emojis that mismatch with their prior sentence context, but which are not explicitly or conventionally ironic; we hypothesize that these will elicit enhanced N400 and/or LFP effects, as the meaning of these non-ironic (but semantically incongruent) emojis would be harder to access and integrate in the surrounding context or generate a still-plausible prediction error. Moreover, as variability and ambiguity have been demonstrated in emoji interpretation (e.g., [12, 13, 14]), we also expected across-participant interpretations of the emojis in this experiment to vary. To this extent we also investigated how individual differences in interpretation mapped on to brain responses elicited during real-time processing.

## Experiment 1

### Method

#### Participants

Forty monolingual English-speaking university students participated. Participants were right-handed [42], and reported no history of neurological impairment or trauma nor use of psychoactive medication. Five datasets were excluded. One dataset was excluded for a high trial rejection rate due to excessive artifact in the EEG (> 20%), one was excluded for an issue with the collection of results of the behavioral task, and two for an inability to get impedances below 10 kΩ. One additional participant felt dizzy near the end of the task and did not complete the experiment. The final dataset consisted of 35 participants (22 female (self-reported gender); *M* = 19.97 years, range: 18–23). Participants provided written informed consent and received either course credit or a small amount of cash. This research was approved by the University of Illinois at Urbana-Champaign Institutional Review Board.

#### Materials

**Fig 1:**
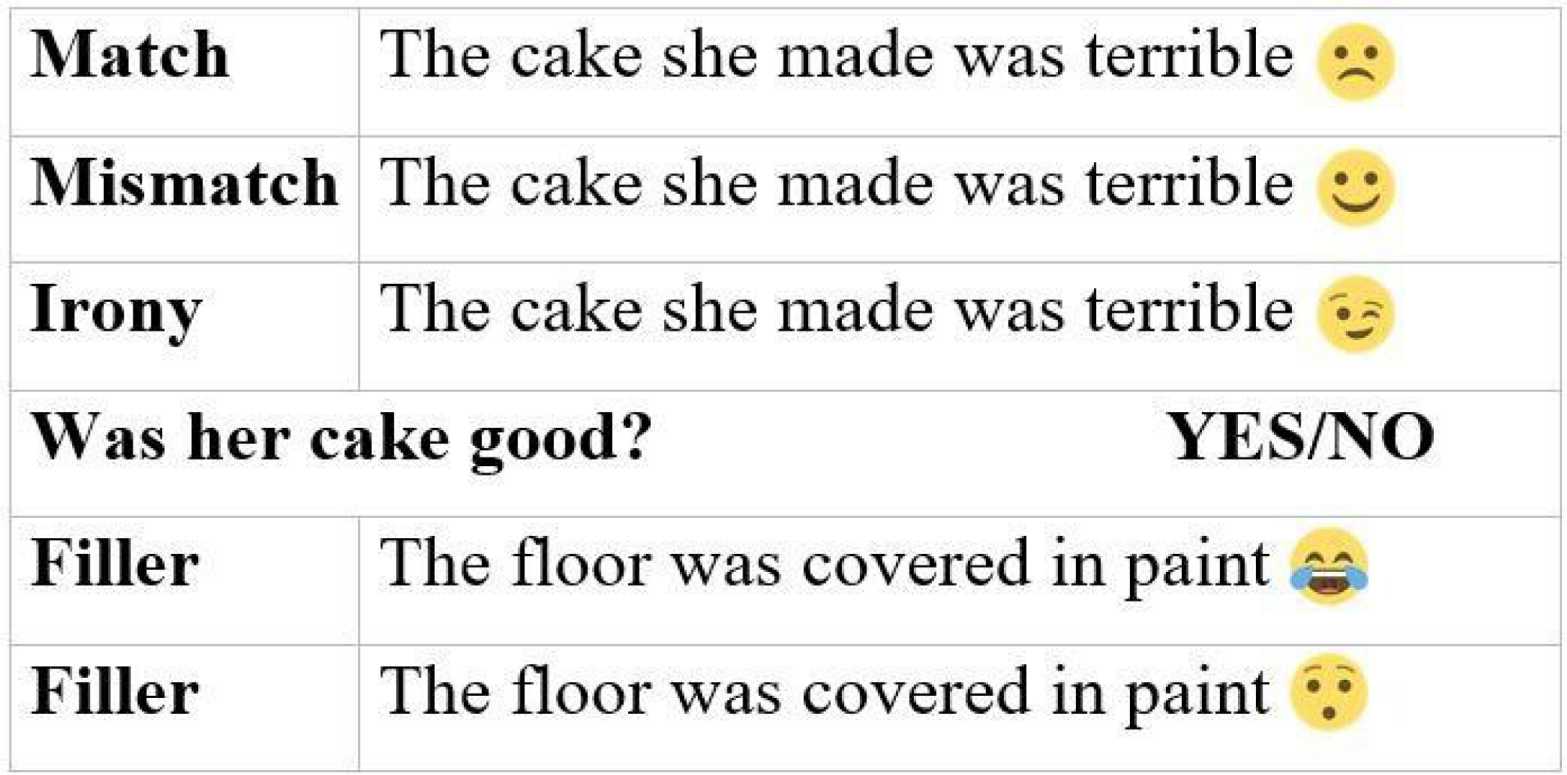
Example stimuli and experimental conditions. Example sentences and a comprehension question in each of the three critical conditions and the two filler conditions.

All stimuli were short English sentences followed by an emoji, to which ERP brain responses were time-locked. The critical emojis were a smile emoji, frown emoji, and wink emoji; filler emoji were a laughing tears emoji or surprised emoji (see Fig 1). The emojis used in the experiment came from EmojiOne (http://emojione.com). The three conditions of critical stimuli were: match, in which the valence of the emoji matched the valence of the sentence (i.e., positive sentence + smile, negative sentence + frown), mismatch, in which the valence of the emoji mismatched the valence of the sentences (i.e., positive + frown, negative + smile), and irony, in which either a positive or negative sentence was followed by a wink emoji. Filler stimuli were non-valenced sentences followed by one of the two filler emojis. Yes/No comprehension questions followed 33% of trials, equally distributed across experimental conditions. Examples of match, mismatch, and irony condition sentences, respectively, an example follow-up question, and filler sentences are provided in Fig 1.

The 180 critical stimuli were counterbalanced in a Latin Square design and distributed across three lists, such that every participant saw every sentence frame only once and, across participants, an equal number of people saw each sentence followed by each of the three emojis. Each list contained 90 positive, 90 negative, and 60 filler (neutral valence) sentences. The 180 valenced sentences were divided evenly so that each list contained 60 sentences in each condition (match, mismatch, irony). This resulted in a total of 30 valence + condition sentences for each combination (i.e., positive match, positive mismatch, positive irony, negative match, negative mismatch, negative irony), per list. Sixty filler sentences were used as well, making for a total of 240 sentences.

A two-stage norming study was carried out to confirm that sentences were being interpreted as predicted. In stage one, participants (*N* = 47, recruited from Amazon Mechanical Turk) were presented with a sentence and asked to rank the three critical emojis (smile, frown, wink) according to their appropriateness with a given sentence. The expected ordering for every sentence would be: match, irony, mismatch. Only stimuli whose mean rank orders matched the expected ordering were used in the experiment; the average ranking (1–3 where 1 is the top-ranked) across all stimuli in each condition followed the expected pattern for match (*M* = 1.15, *SD* = .16), irony (*M* = 2.07, *SD* = .17), and mismatch (*M* = 2.77, *SD* = .20). In stage two, participants (*N* = 120, recruited from Amazon Mechanical Turk) were presented with a sentence followed by one of the three critical emojis and asked to give a 1 (not sarcastic) - 5 (highly sarcastic) judgment on how sarcastic the sentence was. This was done to confirm that irony condition sentences were being rated highly (*M* = 3.43, *SD* = .35) and match condition sentences were being rated lowly (*M* = 1.37, *SD* = .21). Interestingly, mismatch condition sentences were also rated highly for irony (*M =* 3.60, SD = .35). Filler sentences were generally rated as not sarcastic (*M* = 1.63, *SD* = .69). A total of 220 sentences– 110 of each valence–were normed; any sentences that did not meet expectations were removed and further trimming was done until the final set of 90 positive and 90 negative sentences was chosen. Norming statistics reported here reflect ratings for only those items included in the final experiment.

#### Procedure

Participants were tested in a single session lasting approximately 1.25–1.5 h. Upon arrival to the lab, participants provided informed consent and completed a language background questionnaire and an abridged version of the Edinburgh Handedness inventory. For the ERP task, participants were seated in a comfortable chair approximately 100cm in front of a computer monitor and randomly assigned to an experimental list. Each trial consisted of the following sequence of events. A fixation cross appeared on the screen for 300 ms followed by a blank screen for 200 ms. Sentences were presented one word at a time, with each word remaining in the center of the screen for 300 ms followed by a 200 ms blank screen (500 ms SOA), followed by the sentence-final emoji. Participants were told to relax and minimize movements during sentence presentation, to read the sentences as normally as possible, and to treat the emoji as “part of the meaning of the sentence.” There was no mention of sarcasm or irony.

Yes/no comprehension questions followed 33% of the trials (see Fig 1 for an example). There were equal proportions of questions on match, mismatch, irony, and filler condition sentences. Comprehension questions were asked for two reasons. First, we wanted to ensure participant attention to the task. Second, comprehension questions provided a metric for evaluating whether participants interpreted sentences literally or nonliterally. Responses were coded as “literal” if they coincided with the literal meaning of the sentence (e.g., “no” in the example) or “nonliteral” if they did not coincide with the literal meaning of the sentence (e.g., “yes” in the example).

#### EEG recording and data analysis

Continuous EEG was recorded from 28 tin scalp electrodes in an elastic cap (Electro-cap International), from standard and extended 10–20 locations [43]. Eye movements were monitored with electrodes placed below the left eye (referenced off-line to FP1) and at the outer canthus of each eye (referenced off-line in a bipolar montage). EEG was also recorded at the left and right mastoids, with channels referenced during recording to the left mastoid and re-referenced offline to the algebraic mean of the right and left mastoids. Electrode impedances were held below 10 kΩ. EEG was amplified using a BrainAmpDC bioamplifier system (Brain Products GmbH) and digitized with a 1000-Hz sampling rate and an online analog .016–250 Hz bandpass filter. A 0.1–30 Hz bandpass (12 dB/octave roll-off) was applied to the continuous EEG offline [44].

All data processing was done in the EEGLAB [45] and ERPLAB [46] toolboxes in MATLAB. EEG epochs time-locked to the critical emoji were extracted, beginning 200 ms before and ending 1000 ms after presentation of the emoji. Trials containing blinks, muscle activity, drift, or other artifacts were rejected. For participants with over 25% rejections in at least one condition (n = 19), Independent Component Analysis was run on the data to isolate blink and saccade components. If a blink or saccade component was detected based on visual inspection of the component topography and time course, this component was removed from the data. Artifact-corrected data were then re-screened with any remaining aberrant trials rejected from further analysis. The final proportion of total rejected trials was 5.2%. This percentage did not differ significantly across conditions.

ERP components were operationalized as the mean voltage within a time window. The time windows for the relevant components (P200, P600, LFP) were based on previous literature and visual inspection of the data and are as follows: P200: 175–250 ms; P600: 450–750 ms; LFP: 600–900 ms. A 200 ms pre-stimulus baseline was used. Repeated-measures ANOVAs were used to determine differences between conditions; separate ANOVAs were conducted over midline and lateral sites in order to assess the topographic distribution of effects. Every ANOVA included 3 levels of condition (match, mismatch, irony). Midline electrode site ANOVAs (Fz, Cz, Pz, Oz) included those four levels of anteriority; lateral electrodes were grouped into seven left/right hemisphere pairs: FP1/2, F3/4, FC1/2, C3/4, CP1/2, P3/P4, O1/O2. ANOVAs over these lateral electrodes included two levels of hemisphere and seven levels of anteriority. The Greenhouse-Geisser correction for inhomogeneity of variance was applied to all contrasts involving more than one degree of freedom in the numerator; we report corrected p-values and uncorrected degrees of freedom. Only main effects and interactions involving the factor of condition are reported. When a significant main effect of condition or interaction involving condition and a topographic factor was found, two follow up ANOVAs were run with the relevant two-way contrasts (i.e., one for match vs. irony, one for match vs. mismatch), as our primary questions involved comparing the nature of brain responses elicited by the unexpected emojis to those elicited by the expected (match) condition. The N400 time window (300–450 ms) were also analyzed as described above; however, no significant effects emerged from this time window, so these analyses are not discussed further.

### Results

#### Behavioral results

**Fig 2:**
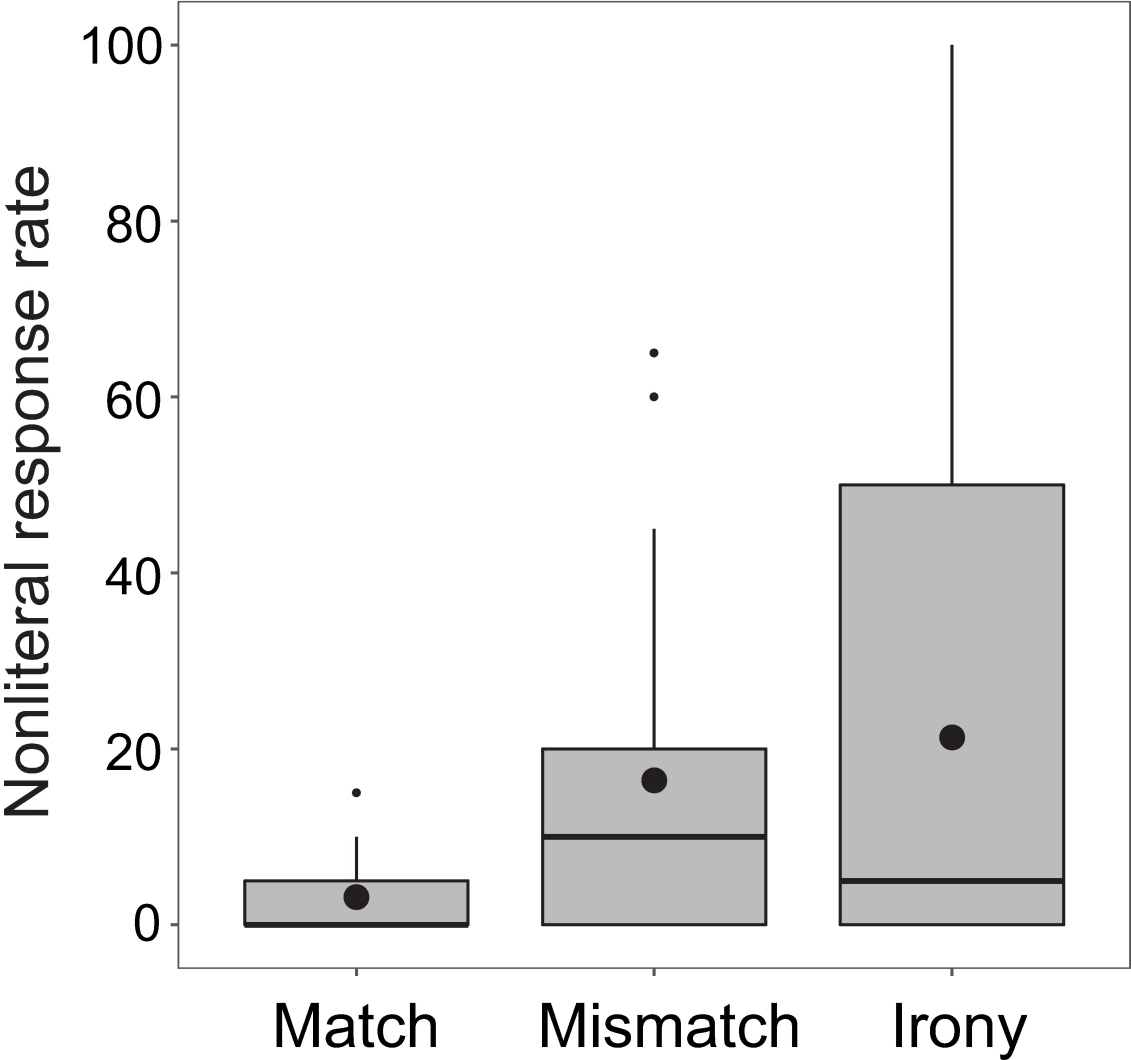
Boxplot of nonliteral response rates in the three conditions in Experiment 1. Horizontal lines in each box depict the median; black circles depict the mean. Boxes extend to first and third quartiles; whiskers extend to 1.5 x interquartile range.

Overall, as seen in Fig 2, there was only a 21.3% nonliteral response rate (SD = 28.3) to prompts in irony condition. The nonliteral response rate in mismatch condition was 16.4% (SD = 18.5); match condition was 3.1% (SD = 4.0).

**Fig 3:**
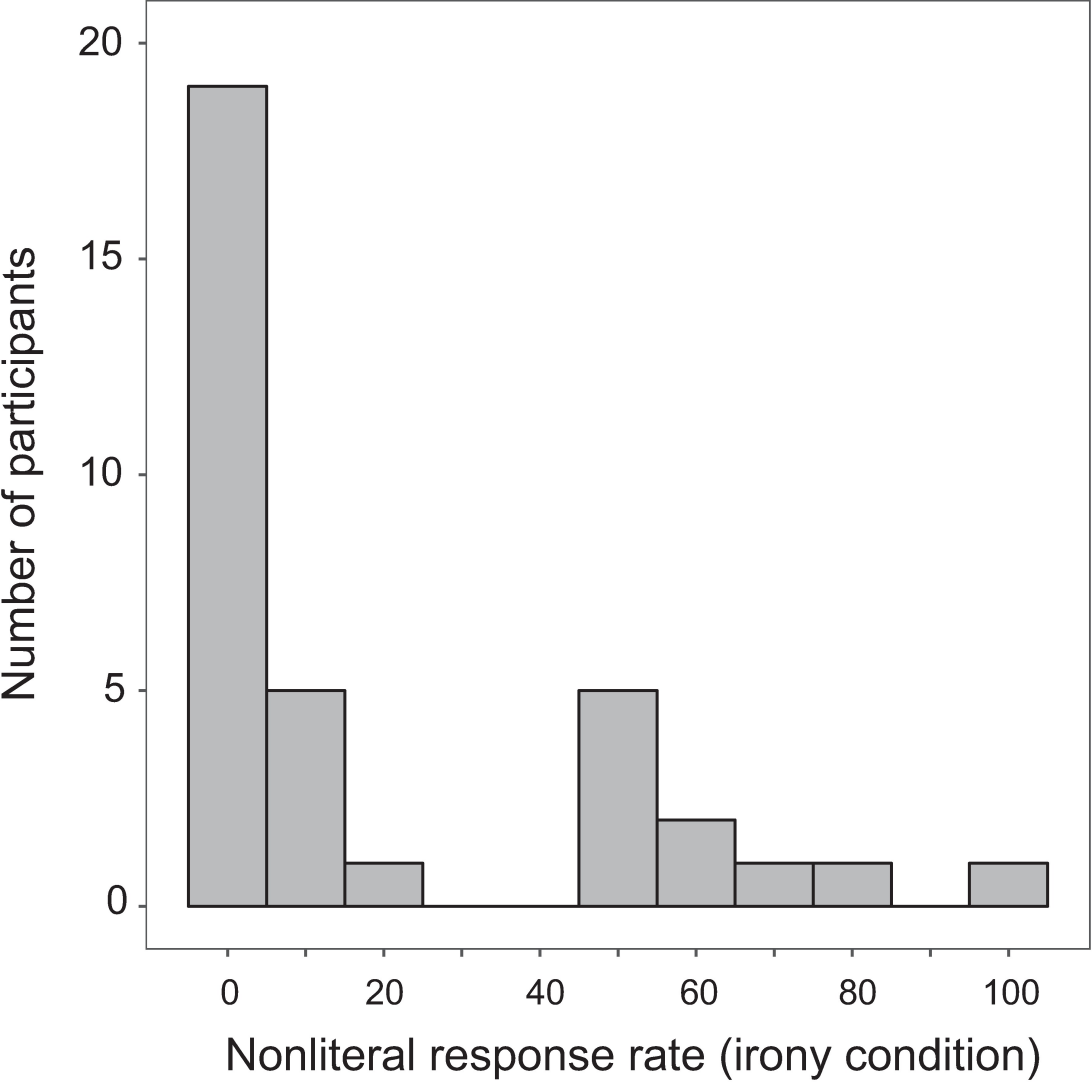
Histogram of nonliteral response rates. Histogram displaying participants’ mean nonliteral response rate in irony condition only.

Fig 3 depicts the distribution of participants’ nonliteral behavioral responses in the irony condition, demonstrating the notable split that existed among participants regarding the behavioral results, specifically in the irony condition. Most participants’ (*n* = 25) response behavior showed no indication of ironic interpretation; these participants responded either uniformly in a literal manner, or with only one or two ironic interpretations during the experiment. The remaining participants (*n* = 10) showed a range of ironic response rates, between 50% and 100%. Thus, in this experiment, participants tended to either comprehend sentences literally, regardless of the emoji, or allowed nonliteral interpretations greater than half the time when presented with a winking emoji.

This distribution suggests that there is a broad, skewed distribution of responders regarding the irony condition in this experiment–those who tend to respond literally and those who allow nonliteral interpretations. Instead of a normal distribution around the mean nonliteral response rate (21%), we see evidence here for one group of participants who were highly consistent with literal responses, and other participants who allowed nonliteral interpretations to varying degrees. To identify whether neural responses were associated with participants’ sentence interpretations, we first divided them into two groups based on the proportion of nonliteral responses: the Literal group (20% and lower, *n* = 25) and the Nonliteral group (50% and higher, *n* = 10).

#### ERP results and discussion

For the nonliteral response group (*n* = 10), analysis in the 175–250 ms time window showed a significant main effect of condition, *F*(2, 18) = 7.02, MSE = 7.84, *p* = .01 at midline sites, *F*(2, 18) = 8.65, MSE = 23.79, *p* = .01 at lateral sites, and a significant interaction between condition and anteriority, *F*(6, 54) = 6.86, MSE = 0.53, *p* < .01 at midline sites, *F*(12, 108) = 4.98, MSE = 1.06, *p* < .001 at lateral sites. Follow up ANOVAs indicate that the main effect of condition was significant between match and irony, *F*(1, 9) = 10.25, MSE = 9.71, *p* = .01 at midline sites, *F*(1, 9) = 11.96, MSE = 32.24, *p* < .01 at lateral sites, and not between match and mismatch, *F*(1, 9) = 1.53 at midline sites, 3.72 at lateral sites. Follow-up ANOVAs indicated a significant condition by anteriority interaction between match and irony, *F*(3, 27) = 12.93, MSE = 0.52, *p* < .001 at midline sites, *F*(6, 54) = 11.58, MSE = 0.88, *p* < .001 at lateral sites, as well as a small condition by anteriority interaction between match and mismatch (*F*(3, 27) = 4.35, MSE = 0.36, *p* = .04) at midline sites only. This indicates that both mismatching and ironic emojis elicited P200 effects, relative to match emojis, and that this effect was frontally-distributed, as is typical for P200 effects. Notably, the P200 was much more reliable and of larger amplitude in the irony condition compared to the mismatch condition.

**Fig 4:**
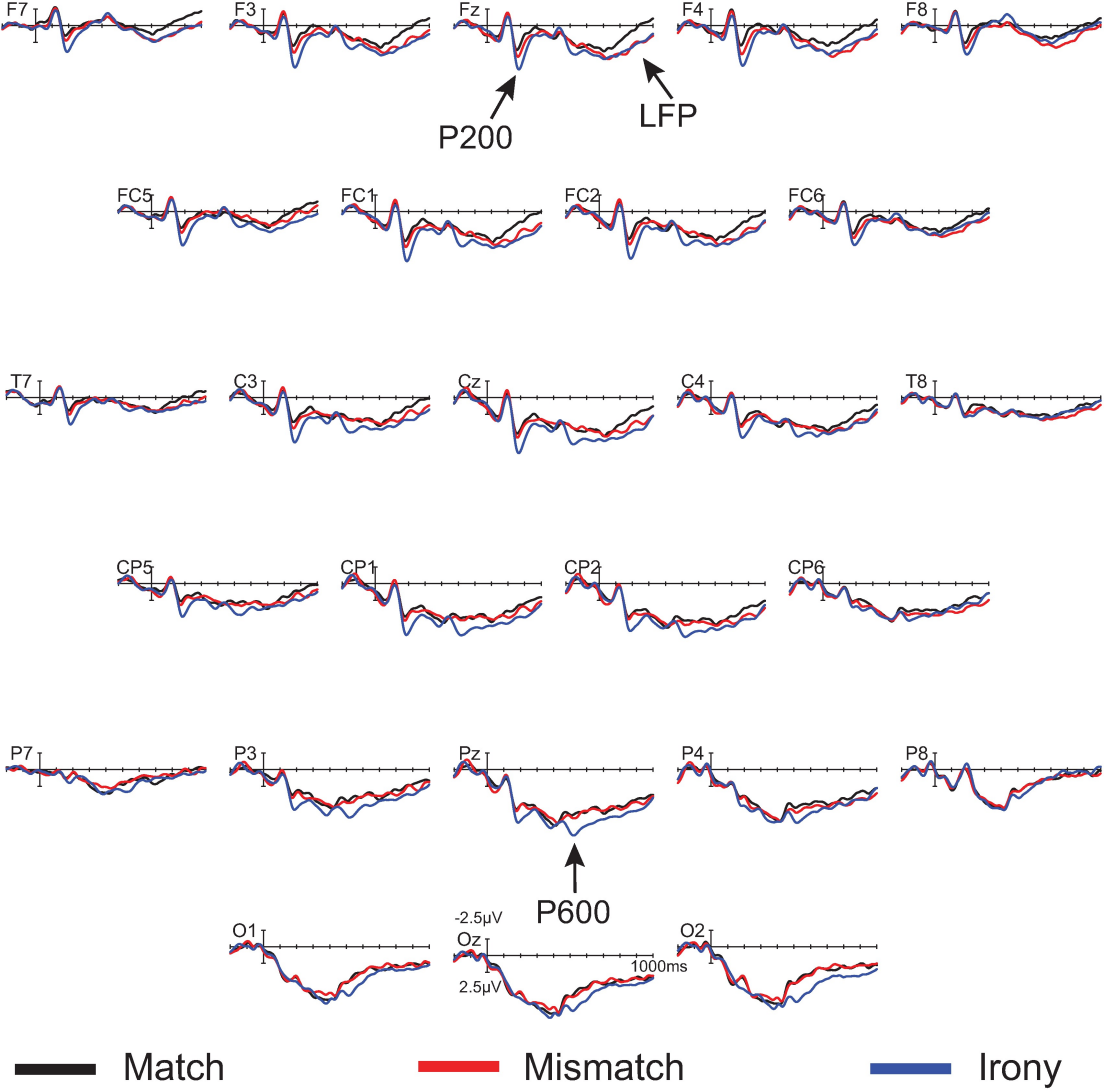
Grand mean ERPs for the nonliteral group (*n* = 10) at 26 representative electrode sites. The vertical calibration bar indicates the temporal onset of the emoji stimulus and extends to indicate ±2.5 μV of brain activity with negative voltage plotted up; 200 ms of prestimulus and 1000 ms of poststimulus brain activity are depicted.

**Fig 5:**
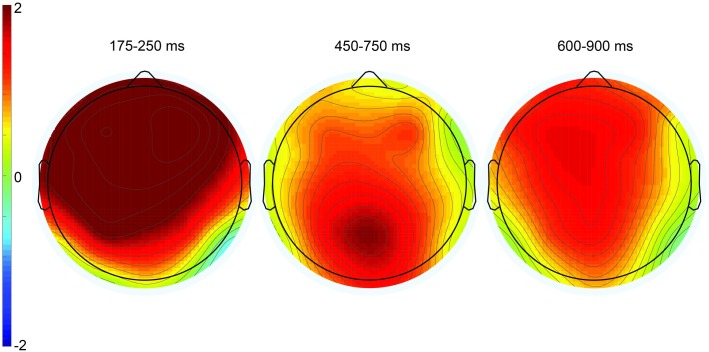
Topographic plots depicting ERP effects from the irony minus match condition difference waves. The three time windows correspond to the P200 component (left), the P600 component (center), and late frontal positivity (right). Color scale (± 2μV) is held constant across scalp maps.

In the 450–750 ms time window, there was a significant effect of condition at midline sites only, *F*(2,18) = 4.41, MSE = 6.23, *p* = .04. Pair-wise follow-up contrasts showed a significant difference between match and irony, *F*(1,9) = 6.43, MSE = 7.23, *p* < .01, and not between match and mismatch, *F*(1, 9) = 0.27. There was no statistically significant interaction, though inspection of the waveforms and topographic plot (see Figs 4 and 5) indicate this effect was strongest over parietal sites.

In the 600–900 ms time window, there was a near-significant main effect of condition *F*(2, 18) = 3.28, MSE = 6.38, *p* = .06 at midline sites, *F*(2, 18) = 3.33, MSE = 15.56, *p* = .06 at lateral sites, but a significant interaction between condition and anteriority, *F*(6, 54) = 3.29, MSE = 0.47, *p* < .01 at midline sites, *F*(12, 108) = 2.46, MSE = 0.73, *p* < .01 at lateral sites. Follow-up ANOVAs reveal a near-significant main effect of condition in the match/irony contrast, *F*(1, 9) = 4.51, MSE = 9.08, *p* = .06 at midline sites, *F*(1, 9) = 4.74, MSE = 23.00, *p* = .06. In the match/mismatch contrast, there was a significant main effect of condition over lateral sites (which include the prefrontal electrodes FP1/2), *F*(1, 9) = 5.08, MSE = 7.71, *p* = .05, but not over midline sites (which do not include prefrontal electrodes) *F*(1,9) = 2.07, MSE = 2.81, *p* = .18. Additionally, the condition by anteriority interaction was significant in the match/mismatch contrast, *F*(3, 27) = 5.65, MSE = 0.44, *p* = .02 at midline sites, *F*(6, 54) = 4.13, MSE = 0.86, *p* = .02. It is apparent from the waveforms and scalp topographies (see Fig 5, right) that this positivity was frontally distributed. Although the LFP was present and of similar amplitude in both the irony and mismatch conditions (see Fig 4), the fact that the effect only neared statistical significance in the match/irony contrast likely results from the small sample size in this group (n = 10); note, however, that this effect is fully replicated both in the nonliteral group in this experiment, as well as in omnibus analyses in Experiments 2 and 3, below. We are therefore confident that this represents a true, replicable effect.

**Fig 6:**
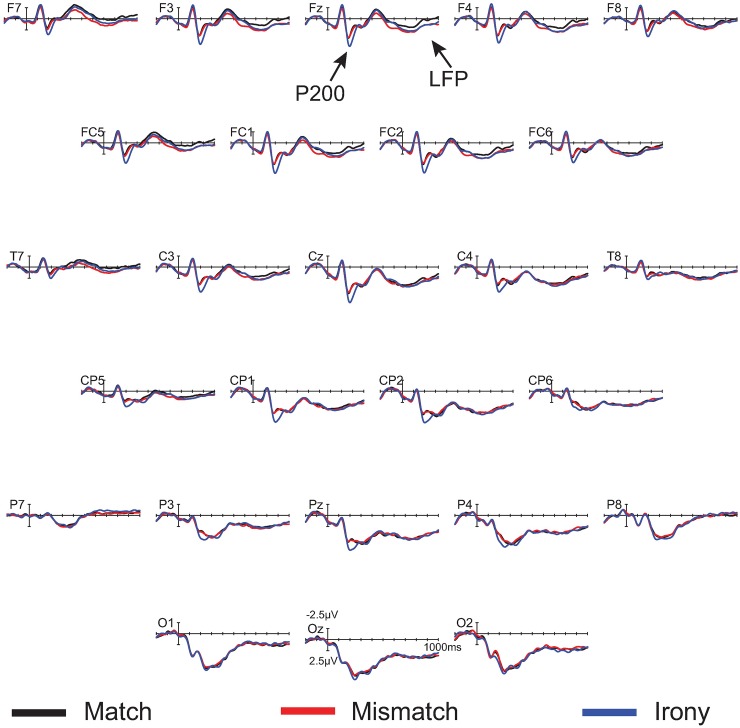
Grand mean ERPs for the literal group (*n* = 25) at 26 representative electrode sites. The vertical calibration bar indicates the temporal onset of the emoji stimulus and extends to indicate 2.5 μV of brain activity with negative voltage plotted up; 200 ms of prestimulus and 1000 ms of poststimulus brain activity are depicted.

**Fig 7:**
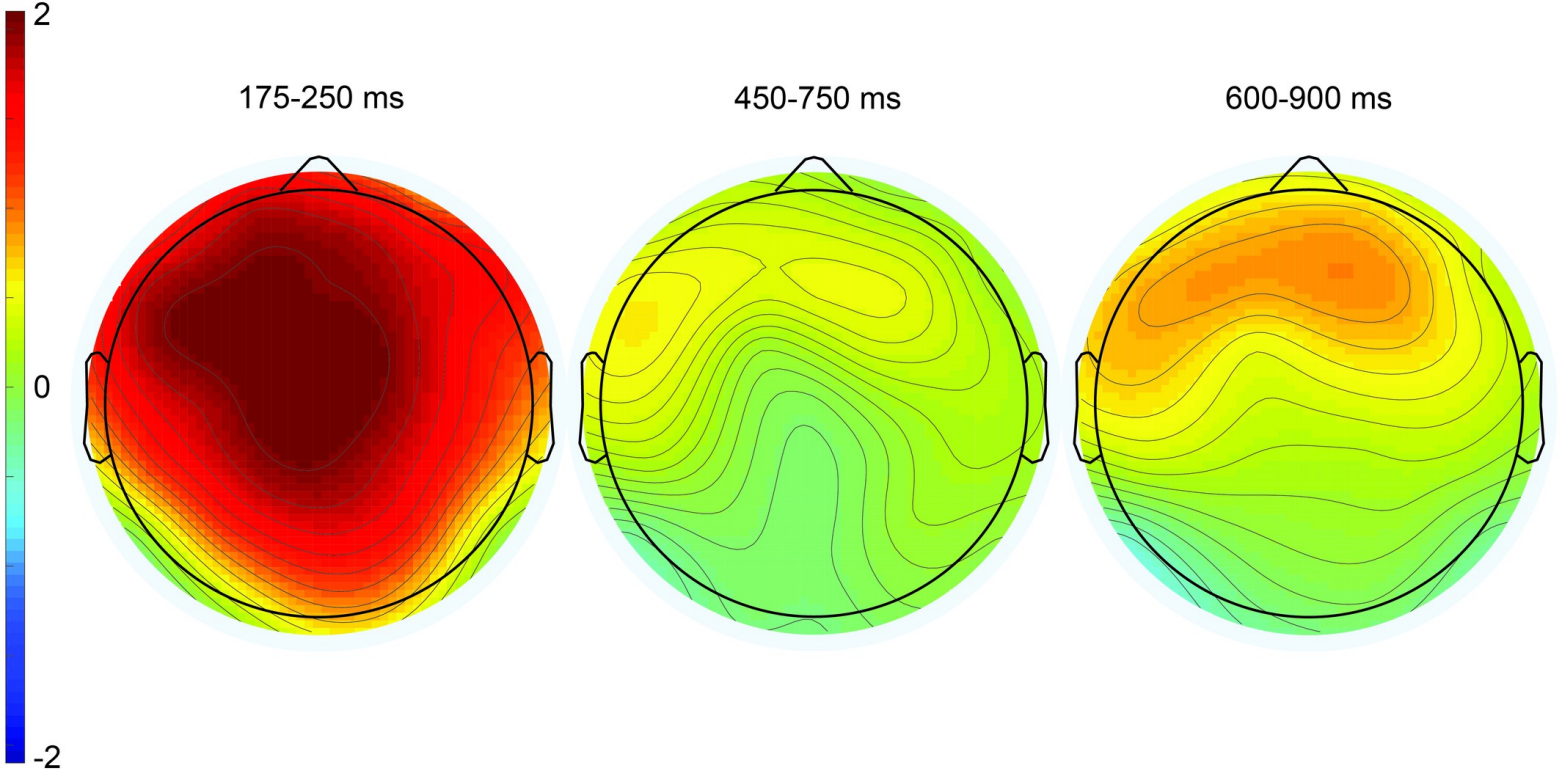
Topographic plots depicting ERP effects from the irony minus match condition difference waves. The three time windows correspond to the P200 component (left), the P600 component (center), and late frontal positivity (right). Color scale (± 2μV) is held constant across scalp maps.

Figs 6 and 7 display the waveforms and topographic maps, respectively, for the literal group.

For participants who responded literally (*n* = 25), there was again a significant main effect of condition in the 175–250 ms time window, *F*(2, 48) = 13.99, MSE = 8.01, *p* < .001 at midline sites, *F*(2, 48) = 13.66, MSE = 25.31, *p* < .001 at lateral sites, and a significant interaction between condition and anteriority, *F*(6, 144) = 9.59, MSE = 0.48, *p* < .001 at midline sites, *F*(12, 288), MSE = 7.77, *p* < .001 at lateral sites. Follow-up ANOVAs showed a significant main effect of condition for the match/irony contrast, *F*(1, 24) = 21.75, MSE = 7.98, *p* < .01 at midline sites, *F*(1, 24) = 21.59, MSE = 25.07, *p* < .001 at lateral sites, as well as a condition by anteriority interaction, *F*(3, 72) = 14.09, MSE = 0.51, *p* < .001 at midline sites, *F*(6, 144) = 11.80, MSE = 0.77, *p* < .001 at lateral sites. Note that there were no effects in the match/mismatch contrasts, *F*s < 1, indicating a frontally-distributed P200 effect elicited selectively by the ironic emojis. These results therefore show a frontally-distributed distributed P200, elicited selectively by ironic emojis.

There was no main effect of condition in the 450–750 ms time window for the literal group. There were significant interactions of condition and anteriority during this time window; however, visual inspection of the waveforms indicates that this reflects the onset of the late frontal positivity, which we analyze fully below.

There was no main effect of condition in the 600–900 ms time window, but there was a significant condition by anteriority interaction, *F*(6, 144) = 3.54, MSE = 0.68, *p* = .02 at midline sites, *F*(12, 288) = 3.00, MSE = 1.18, *p* = .02 at lateral sites. This interaction was significant between match and irony conditions, *F*(3, 72) = 4.33, MSE = 0.60, *p* = .02 at midline sites, *F*(6, 144) = 3.11, MSE = 1.09, *p* < .01 at lateral sites, and between match and mismatch conditions, *F*(3, 72) = 5.00, MSE = 0.84, *p* = .02 at midline sites, *F*(6, 144) = 4.36, MSE = 1.39, *p* = .02 at lateral sites. These results indicate late frontal positivities elicited by both the mismatch and irony emojis.

To further investigate the relationship between interpretation and brain responses, we correlated individual participants’ proportions of nonliteral responses in the irony condition with their P600 effect amplitude, derived from the irony minus match difference wave at Pz. When considering all participants, there was a significant positive correlation between nonliteral response rate and P600 amplitude, *r* = .524, *p* = .001. Because a large subset of participants showed no variability in their behavioral responses (fully literal responders; *n* = 19), we recomputed the correlation coefficient including only those who showed at least some response variability (*n* = 16); the correlation became stronger and remained significant: *r* = .61, *p* = .004. The R^2^ of 0.37 indicates that approximately 37% of the variability in P600 amplitude in our sample of variable responders can be explained by participants’ nonliteral response rate. This robust effect suggests strongly that P600 effects to ironic emojis were driven by reanalysis of the literal meaning of the sentence.

Taken together, the results from this experiment showed that ironic emojis elicited P200 effects across the full sample of participants compared to matching emojis, and more importantly, that ironic emojis elicited P600 effects in the subset of participants who allowed nonliteral interpretations of the sentences in response to the emojis. The P200-P600 complex is the same pattern ERP responses seen in prior reports of verbal irony [20, 21, 22, 23]. However, in our study, the second phase of the complex (the P600) was seen only in participants who allowed the ironic emoji to override the literal meaning of the words in the sentence. This finding was corroborated through the correlation analysis, linking P600 effect amplitudes to the proportion of trials on which participants responded nonliterally. Thus, the results from this experiment support the notion that the P600 may reflect monitoring and/or reanalysis processes associated with updating the conceptual or semantic meaning of the sentence (cf. [28, 29, 30]).

Notably, unlike prior studies on verbal irony, we found an additional late frontal positive-going ERP component, elicited by all unexpected emojis. This was found for both the mismatch and irony emojis, in both the literal and nonliteral responder groups. This late frontal component is highly similar to that seen in ERP studies of sentence comprehension in which participants encounter un-predicted lexical continuations or sentence completions [38, 38, 40, 41]. Here, the mismatching and ironic emojis could be considered unpredicted completions of sentences, which, through their emotional valence, have predictable emoji counterparts (e.g., the matching smiling or frowning faces for the positively- and negatively-valenced sentences, respectively). On the whole, results of Experiment 1 suggest that unexpected and ironic emojis elicit brain responses highly similar to their verbal counterparts.

## Experiment 2

In Experiment 1, we showed that irony-induced emojis elicited a P200-P600 complex in participants who used the emoji to override the literal meaning of the words in the sentence. However, a high proportion of participants did not show any evidence of ironic interpretation of the sentences associated with the wink emoji. This is reasonable, as the stimuli were inherently ambiguous: without any sort of background knowledge or additional context, the wink was the only cue to irony, and many participants may have adopted a default strategy of interpreting sentences literally instead of using the emojis to guide interpretation.

In Experiment 2 we therefore sought to replicate and extend the primary effects elicited in Experiment 1 by alerting participants to the possible presence of irony (which we referred to as “sarcasm” with participants) in the experiment. This experiment used identical materials to Experiment 1; the only difference was the brief mention of sarcasm during the instructions for the task. Through this mention we intended to elicit a greater number of nonliteral interpretation of sentences.

### Method

#### Participants

Thirty-eight monolingual English speakers participated in this experiment for either course credit or monetary compensation. All participants were right-handed and reported no history of brain trauma, neurological impairment, or psychoactive medication; in addition, none of the participants participated in Experiment 1. Three datasets were excluded for high trial rejection rate (> 20%), making a final dataset of 35 participants (24 female (self-reported gender); *M* = 20.49 years, range: 18–25). The same consent and compensation protocol was used as in Experiment 1.

#### Materials

The same materials and lists were used as in Experiment 1.

#### Procedure

The same procedure was used as in Experiment 1. The only difference was an additional instruction at the beginning of the experiment: participants were told, “This study is investigating sarcasm… Some of the emojis you see will be sarcastic.” Participants were not told specifically that wink emojis should be interpreted sarcastically; rather, what emojis and sentences they should treat as sarcastic was left entirely up to them.

#### Recording and data analysis

The same recording and data analysis techniques were used as in Experiment 1.

### Results

#### Behavioral results

**Fig 8:**
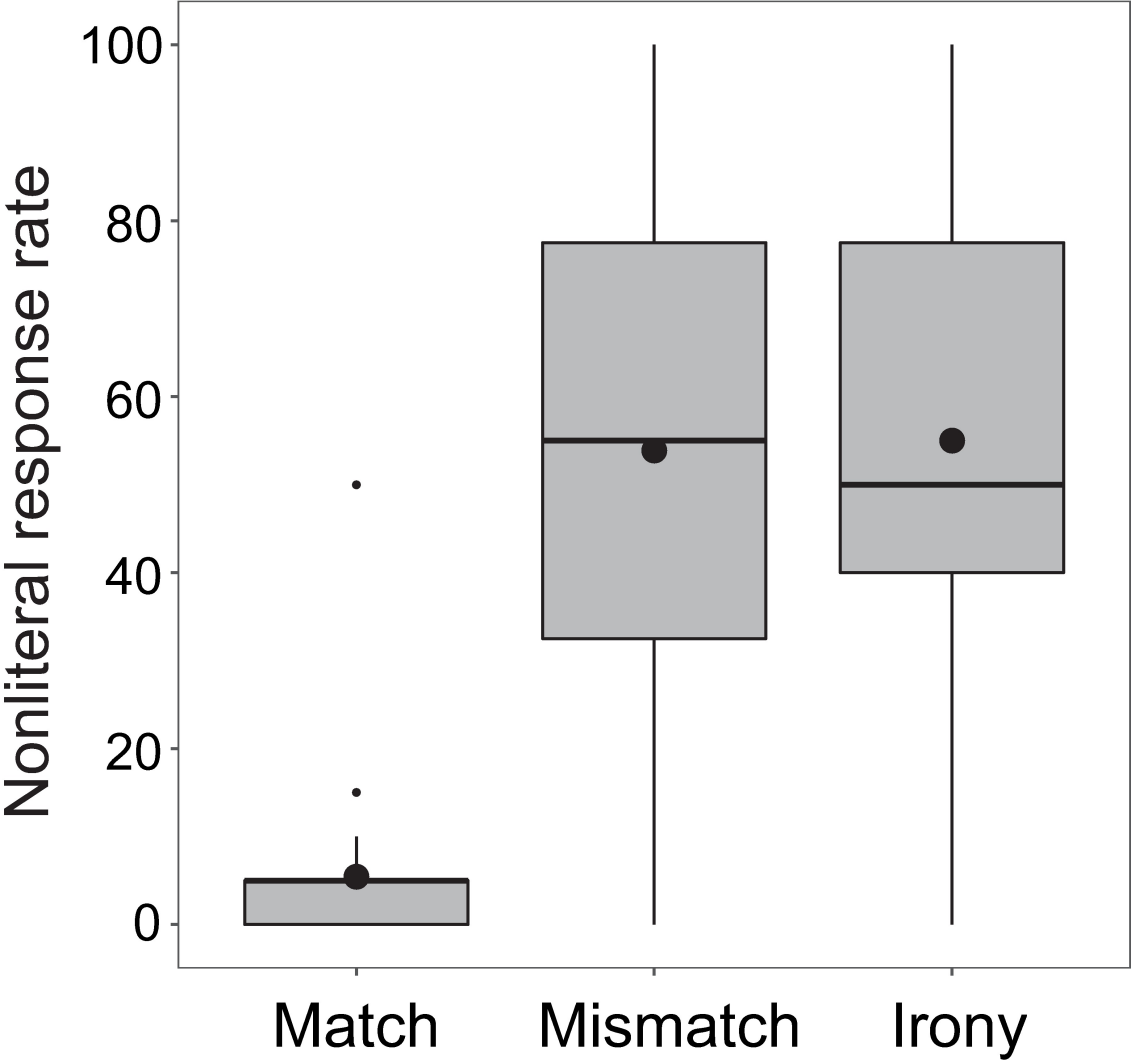
Boxplot of nonliteral response rates in the three conditions in Experiment 2. Horizontal lines in each box depict the median; black circles depict the mean. Boxes extend to first and third quartiles; whiskers extend to 1.5 x interquartile range.

As is evident from Fig 8, the additional instruction achieved the desired effect, as nonliteral response rate in irony condition rose from 21.3% in Experiment 1 to 55.0% in Experiment 2 (SD = 30.4), though there was still considerable between-subject variability in nonliteral response rate. Since winks were not specified as the marker of irony, the nonliteral response rate in mismatch condition rose as well, from 16.4% in Experiment 1 to 53.9% in Experiment 2 (SD = 28.9). Match condition stayed low, increasing slightly from 3.1% in Experiment 1 to 5.4% in Experiment 2 (SD = 9.1).

#### Grand Mean ERP results and discussion

There was a significant main effect of condition in the 175–250 ms time window, *F*(2, 68) = 14.47, MSE = 6.17, *p* < .001 at midline sites, *F*(2, 68) = 12.82, MSE = 20.98, *p* < .001 at lateral sites, as well as reliable interactions between condition and anteriority, *F*(6, 204) = 6.33, MSE = .68, *p* < .001 at midline sites, *F*(12, 408) = 4.10, MSE = 1.09, *p* < .001 at lateral sites. Follow up contrasts between the match and irony conditions showed a reliable main effect of condition, *F*(1, 34) = 17.91, MSE = 8.32, *p* < .001 at midline sites, *F*(1, 34) = 16.60, MSE = 27.43, *p* < .001 at lateral sites, and interaction between condition and anteriority, *F*(3, 102) = 9.43, MSE = 0.73, *p* < .001 at midline sites, *F*(6, 204) = 5.70, MSE = 1.31, *p* = .01 at lateral sites, as well as an interaction between condition and hemisphere over lateral sites, *F*(1, 34) = 5.96, MSE = 0.93, *p* = .02. This additional interaction with hemisphere indicates that the positivity had a slightly left hemisphere preponderance, though it should be noted that the P200 still showed a typical fronto-central preponderance. There were no significant effects for the match/mismatch contrast, *F*s < 1.

**Fig 9:**
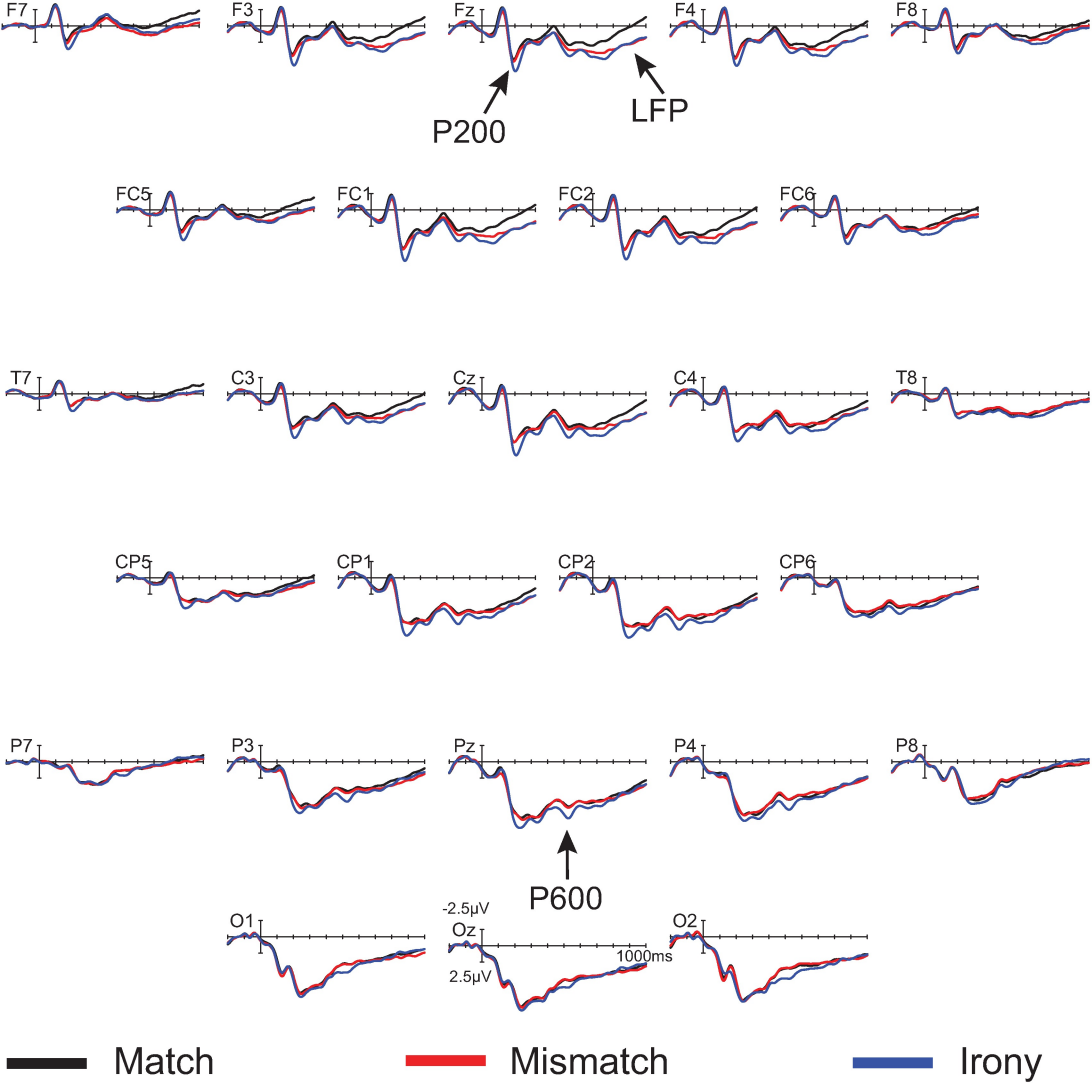
Grand mean ERPs in Experiment 2 (*n* = 35) at 26 representative electrode sites. The vertical calibration bar indicates the temporal onset of the emoji stimulus and extends to indicate 2.5 μV of brain activity with negative voltage plotted up; 200 ms of prestimulus and 1000 ms of poststimulus brain activity are depicted.

**Fig 10:**
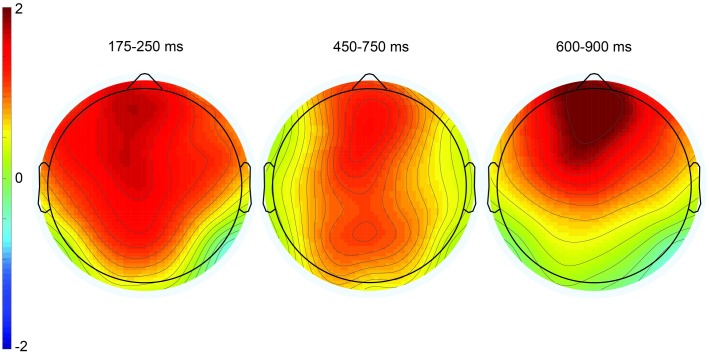
Topographic plots depicting ERP effects from the irony minus match condition difference waves. The three time windows correspond to the P200 component (left), the P600 component (center), and late frontal positivity (right). Color scale (± 2μV) is held constant across scalp maps.

In the 450-750ms time window there was a significant main effect of condition, *F*(2, 68) = 8.37, MSE = 5.49, *p* < .001 at midline sites, *F*(2, 68) = 7.71, MSE = 17.17, *p* < .01 at lateral sites, and a significant condition by anteriority interaction, *F*(6, 204) = 4.98, MSE = 0.75, *p* < .001 at midline sites, *F*(12, 408) = 4.19, MSE = 1.24, *p* = .001 at lateral sites. Follow-up contrasts for the match/irony comparison showed a main effect of condition, *F*(1, 34) = 15.96, MSE = 5.48, *p* < .001 at midline sites, *F*(1, 34) = 19.27, MSE = 13.54, *p* < .001, and an interaction between condition and anteriority, *F*(3, 102) = 6.55, MSE = 0.76, *p* < .01 at midline sites. As can be seen in Figs 9 and 10, this interaction was driven by the presence of a small P600 effect at parietal electrode sites (e.g., CP1, CP2, P3, Pz, P4, where P600 effects are typically largest). The condition and anteriority interaction was significant as well, but this is due to as the onset of the late frontal positivity over more anterior electrode sites and we discuss this in the following paragraph. Notably the correlation between nonliteral response rate and P600 effect amplitude at Pz was significant, r = 0.39, *p* = .02, as in Experiment 1.

In the 600–900 ms time window, there was a significant main effect of condition *F*(2, 68) = 6.77, MSE = 5.41, *p* < .01 at midline sites, *F*(2, 68) = 7.97, MSE = 18.05, *p* < .001) at lateral sites, and interaction between condition and anteriority, *F*(6, 204) = 10.90, MSE = 0.85, *p* < .001 at midline sites, *F*(12, 408) = 8.44, MSE = 1.46, *p* < .001 at lateral sites. Pair-wise contrasts showed a significant effect of condition for the match/irony contrast, *F*(1, 34) = 11.99, MSE = 5.97, *p* = .001 at midline sites, *F*(1, 34) = 7.97, MSE = 18.05, *p* < .001 at lateral sites, and it showed a frontal distribution (condition by anteriority interaction: *F*(3, 102) = 22.02, MSE = 0.71, *p* < .001 at midline sites, *F*(6, 204) = 13.99, MSE = 1.32, *p* < .001 at lateral sites). The effects were qualitatively similar for the match/mismatch contrast (condition by anteriority interaction: *F*(3, 102) = 10.71, MSE = 0.99, *p* < .001 at midline sites, *F*(6, 204) = 10.45, *p* < .001 at lateral sites).

Overall, Experiment 2 replicated and extended the findings from Experiment 1. By mentioning the possibility of sarcasm briefly during the instructions, a greater number of participants showed nonliteral interpretations of the sentences with emojis. As with both groups in Experiment 1, we found a P200 effect, and as in the nonliteral group in Experiment 1, this effect followed by a P600 effect in response to the ironic (wink) emoji in Experiment 2. Importantly, this effect was found not only in a restricted group of participants who allowed nonliteral responses, but indeed in the grand mean. Again, as in Experiment 1, P600 effect amplitudes over posterior sites were correlated with nonliteral response rates. Taken with the grand mean results, this shows that individual variability in nonliteral interpretations was still a significant driver of the P600 effect seen over posterior sites; by boosting the overall nonliteral response rate, our mention of sarcasm to participants in the instructions made this effect systematic enough to surface in the grand mean ERPs.

Additionally, we replicated the late frontal positivity elicited by both types of unexpected emojis (mismatch and ironic). Note that this frontal positivity showed some temporal overlap with the posteriorly distributed P600, leading to the broadly distributed topography for the positivity in the 450–750 ms time window compared to the clear frontal distribution in the 600–900 ms time window (center and right scalp plots in Fig 10). Nonetheless, the fact that nonliteral response rates in the 450–750 ms time window were correlated significantly with effect amplitudes at Pz, as mentioned above, more so than at the frontal electrode site Fz (r = .28, *p* = .10), indicates that nonliteral sentence interpretations associated with emoji-related irony were indexed by this posteriorly distributed P600.

Finally, it is important to note that no P200 or P600 effects were found in the mismatch condition, despite a similarly high overall nonliteral comprehension question response rate in this condition, and nonliteral response rates were not correlated with P600 effect amplitudes in this condition (r = .10, *p* = .58). However, there was still a reliable late frontal positivity in this condition, signaling that the mismatching emojis were nonetheless unexpected and unpredicted. Two non-mutually exclusive explanations for this lack of a P600 effect readily come to mind. First, ERP components like the P600 only index cognitive processes that are time- and phase-locked to the onset of the critical stimulus. It is possible that, given its broad, conventional use to signal irony/sarcasm [15, 16, 17], the reanalysis or reinterpretation processes giving rise to nonliteral interpretations in the mismatch condition are more variable in their time course than those involved in the irony condition. The high conventionality of the wink emoji to signal irony may result in a more consistent time-locking of reanalysis processes to the onset of the emoji stimulus. The second possibility has to do with the informativity of the emojis used within the context of the experiment. The wink emoji was only present in the irony condition, whereas the smiley and frowny faced emojis were counterbalanced across the match and mismatch conditions. Thus, within the experimental context, the wink was consistent in its relation with irony, whereas the smiley and frowny faced emojis were inconsistent: they could occur both in the match condition, or as mismatching (unexpected) stimuli. This direct mapping between the wink and irony and indirect mapping between the smile and frown and irony within the experimental context may be partly responsible for the pattern of effects both in this experiment and Experiment 1. Experiment 3 was designed to follow-up on this possibility.

## Experiment 3

Experiment 3 was designed to alleviate a potential emoji-to-condition mapping confound present in the first two experiments. In those experiments, the irony condition was always a wink, but the match and mismatch could be either a smile or a frown. In Experiment 3, only negatively valenced sentences were used (plus the neutral fillers), resulting in a 1:1 mapping between condition and emoji for the critical match, mismatch, and irony conditions. The frown emoji was always a match, smile always a mismatch, and wink always irony.

### Method

#### Participants

Thirty-eight monolingual English speakers participated in this experiment for either course credit or monetary compensation. All participants were right-handed and reported no history of brain trauma, neurological impairment, or psychoactive medication; in addition, none of the participants participated in Experiment 1 or 2. Two datasets were excluded for high trial rejection rate (> 20%), making a final dataset of 36 participants (28 female (self-reported gender); *M* = 20.4 years, range: 18–35). The same consent and compensation protocol was used as in Experiments 1 and 2.

#### Materials

The same lists were used as in Experiment 1 and 2 except all positively valenced sentences were removed. Each participant in this experiment saw 150 sentences: 30 match, 30 mismatch, and 30 irony, plus 60 filler sentences. The percentage of trials with comprehension questions in each condition was raised to 50%.

#### Procedure

The same procedure was used as in Experiment 1 and 2. Due to the experiment having fewer stimuli, these sessions took around 1–1.25 h. Participants were still told that the experiment was generally investigating sarcasm, like in Experiment 2.

#### Recording and data analysis

The same recording and data analysis techniques were used as in Experiments 1 and 2.

### Results

#### Behavioral results

**Fig 11:**
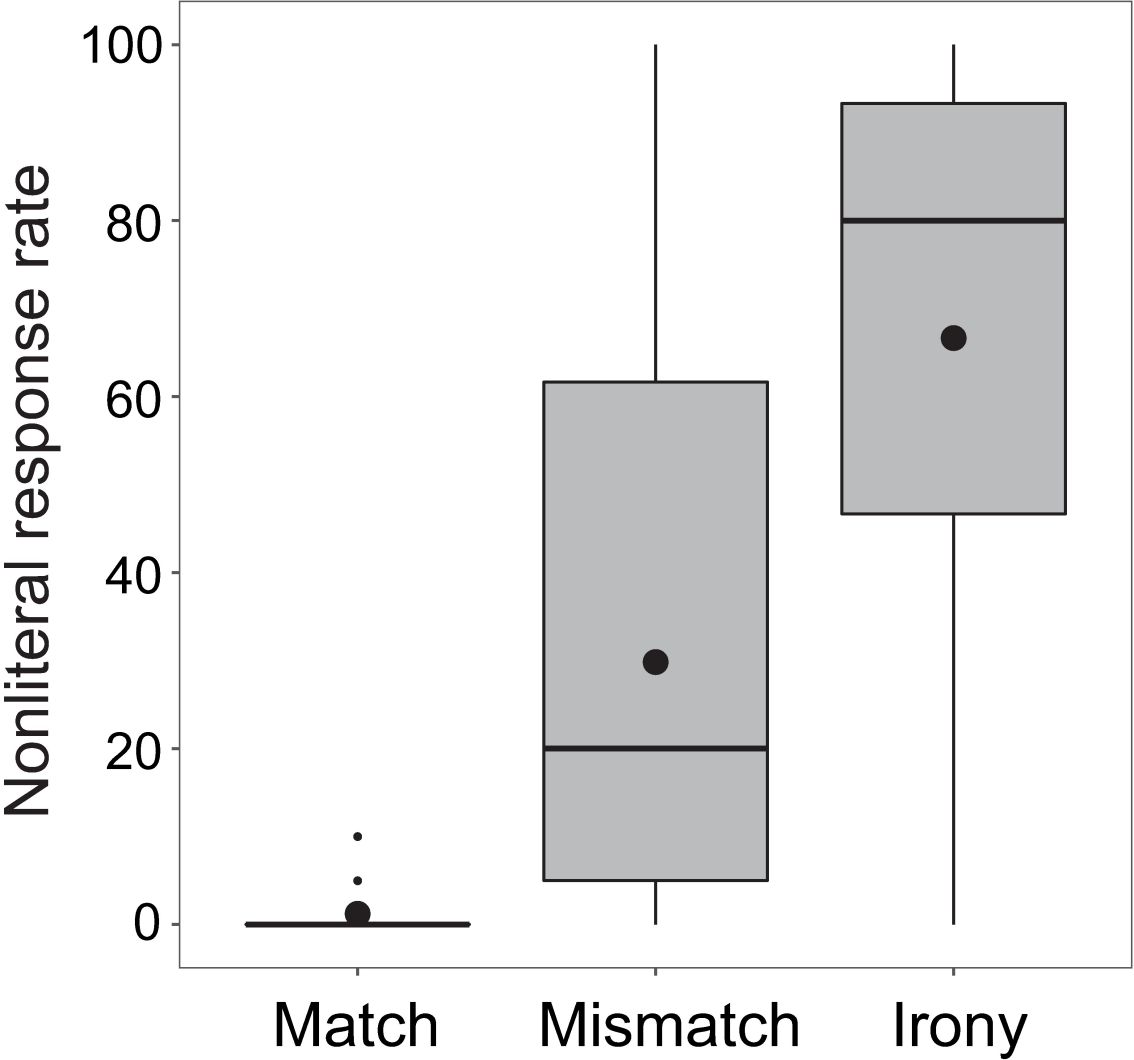
Boxplot of nonliteral response rates in the three conditions in Experiment 3. Horizontal lines in each box depict the median; black circles depict the mean. Boxes extend to first and third quartiles; whiskers extend to 1.5 x interquartile range.

In Experiment 3, irony condition had a nonliteral response rate of 66.6% (SD = 32.5), mismatch condition had a nonliteral response rate of 29.8% (SD = 31.2), and match condition had a nonliteral response rate of 1.2% (SD = 2.7). Fig 11 shows the by-condition response rates for Experiment 3.

#### Grand Mean ERP results and discussion

**Fig 12:**
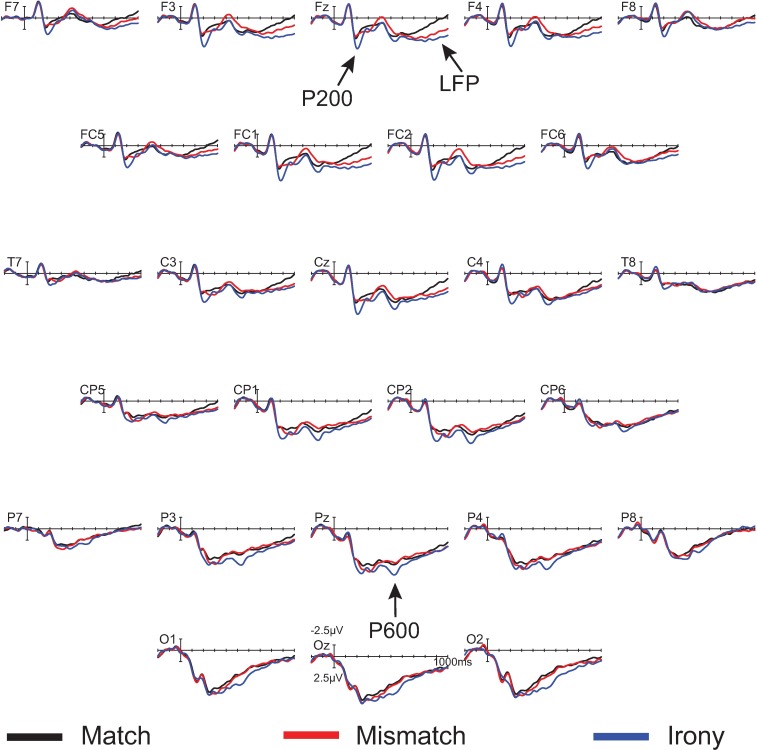
Grand mean ERPs in Experiment 3 (*n* = 36) at 26 representative electrode sites. The vertical calibration bar indicates the temporal onset of the emoji stimulus and extends to indicate 2.5 μV of brain activity with negative voltage plotted up; 200 ms of prestimulus and 1000 ms of poststimulus brain activity are depicted.

**Fig 13:**
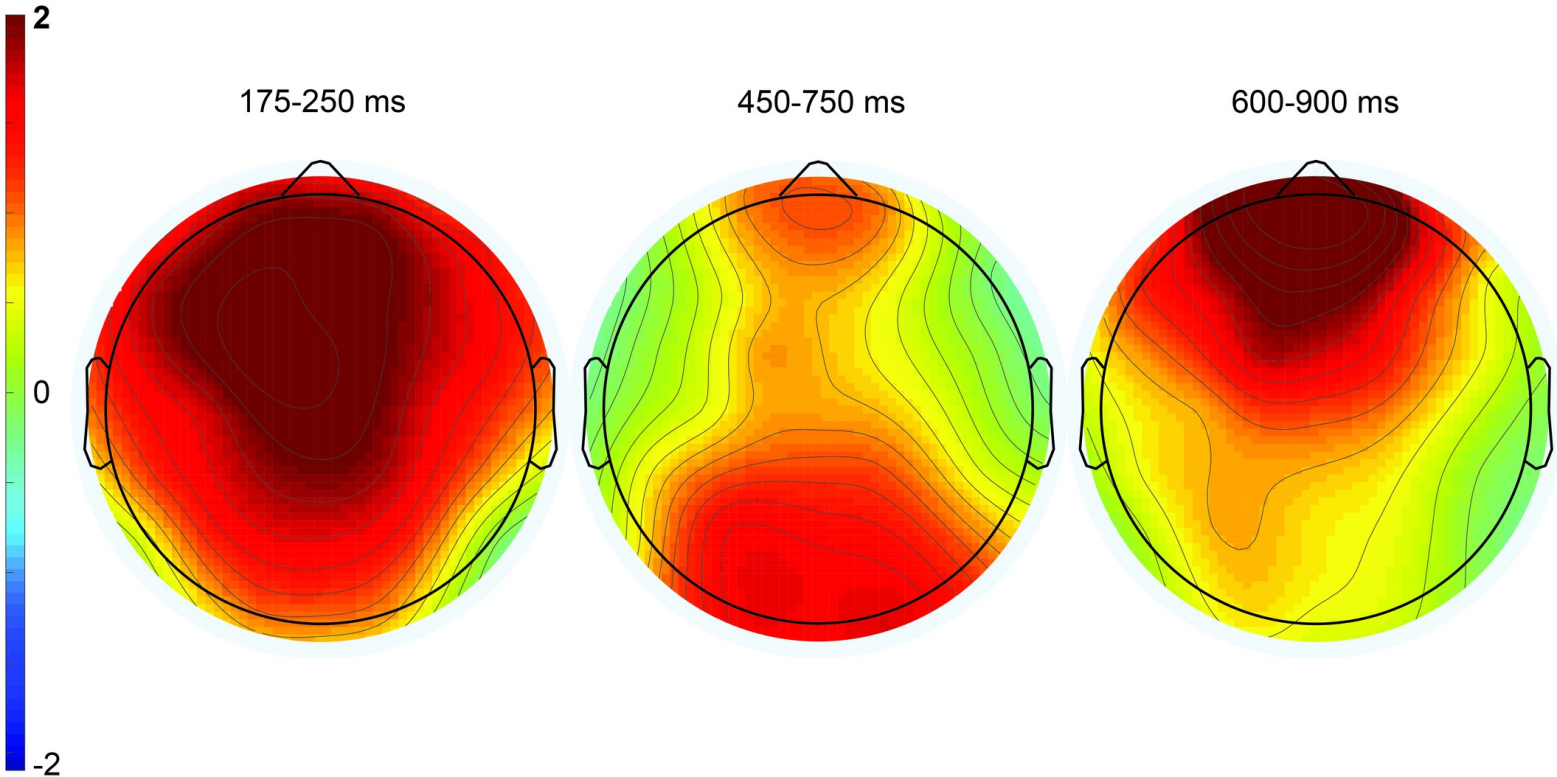
Topographic plots depicting ERP effects from the irony minus match condition difference waves. The three time windows correspond to the P200 component (left), the P600 component (center), and late frontal positivity (right). Color scale (± 2μV) is held constant across scalp maps.

Figs 12 and 13 display the waveforms and topographic maps, respectively, from Experiment 3.

There was a significant main effect of condition in the 175–250 ms time window, *F*(2, 70) = 15.39, MSE = 11.23, *p* < .001 at midline sites, *F*(2, 70) = 18.54, MSE = 31.64, *p* < .001 at lateral sites, as well as a condition by anteriority interaction, *F*(6, 210) = 5.81, MSE = 1.42, *p* < .001 at midline sites, *F*(12, 420) = 4.65, MSE = 2.35, *p* < .01 at lateral sites. Pair-wise contrasts showed that these effects were significant for the match/irony comparison (main effect of condition: *F*(1, 35) = 24.72, MSE = 11.47, *p* < .001 at midline sites, *F*(2, 70) = 28.50, MSE = 32.06, *p* < .001 at lateral sites; condition by anteriority interaction: *F*(3, 105) = 6.77, MSE = 1.37, *p* < .01 at midline sites, *F*(6, 210) = 5.14, MSE = 2.11, *p* = .01 at lateral sites), but not for the match/mismatch comparison, *F*s < 1. As in the prior experiments, this indicates an enhanced frontally-distributed P200 effect elicited by the irony emojis, but not the mismatch emojis.

In the 450–750 ms time window, there was a significant main effect of condition, *F*(2, 70) = 10.69, *p* < .01 at midline sites, *F*(2, 70) = 6.40, MSE = 32.51, *p* < .01 at lateral sites. Pairwise comparisons indicate this main effect was significant between match and irony, *F*(1, 35) = 6.90, MSE = 12.85, *p* = .01 at midline sites, *F*(1, 35) = 5.90, MSE = 39.23, *p* = .02 at lateral sites, and not between match and mismatch, *F*s < 1.

In the 600–900 ms time window, there was a significant main effect of condition, *F*(2, 70) = 5.15, MSE = 8.79, *p* < .01 at midline sites, *F*(2, 70) = 6.86, MSE = 28.17, *p* < .01 at lateral sites, and interaction between condition and anteriority, *F*(6, 210) = 4.64, MSE = 1.63, *p* < .001 at midline sites, *F*(12, 420) = 4.94, MSE = 2.60, *p* < .001, at lateral sites. Pair-wise comparisons showed that the main effect of condition was significant when comparing match and irony completions, *F*(1, 35) = 9.25, MSE = 9.85, *p* = .001 at midline sites, *F*(1, 35) = 12.56, MSE = 30.04, *p* = .001 at lateral sites, and the condition by anteriority interaction was significant for both match/irony and match/mismatch contrasts (match/irony contrast: *F*(3, 105) = 8.44, MSE = 1.32, *p* < .001 at midline sites, *F*(6, 210) = 9.24, MSE = 2.45, *p* < .001 at lateral sites; match/mismatch contrast: *F*(3, 105) = 6.56, MSE = 1.68, *p* < .001 at midline sites, *F*(6, 210) = 5.44, MSE = 2.50, *p* < .01 at lateral sites).

These results further replicate the findings of P600 effects elicited the wink (ironic) emoji, and LFP effects eleicited by both unexpected emoji types (ironic and mismatch). Most importantly, there was still a P200 effect elicited selectively by the wink emoji. This is despite the fact that the design of this experiment removed the informativity confound present in the prior experiments. In this experiment, the ironic (wink) and mismatch (smile) emojis were equally informative, in that there was a one-to-one mapping between condition and emoji within the experimental context. This suggests that the P200 effect found in response to the ironic emojis in all three experiments was not a product of experiment-internal informativity of the wink stimulus.

### Cross-experiment correlation analysis

**Fig 14:**
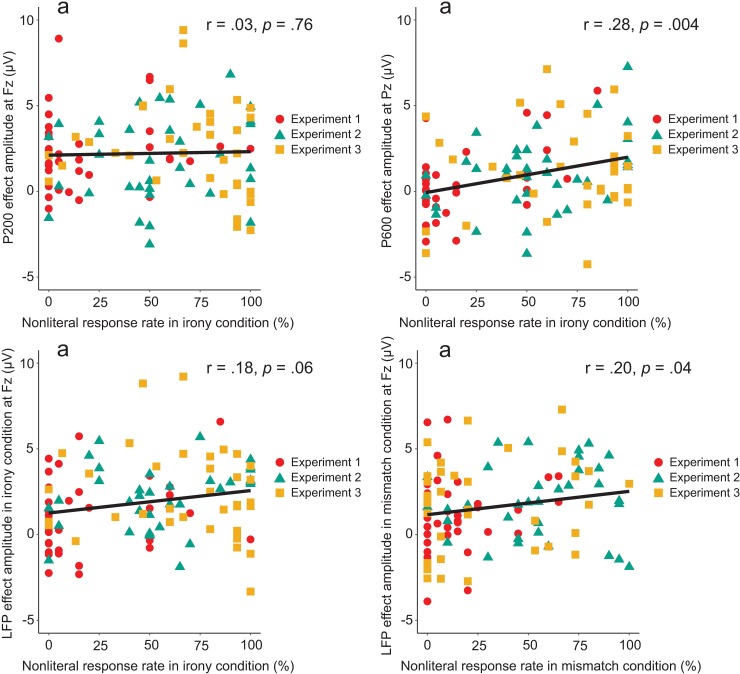
Correlations of behavioral responses with ERP components across all three experiments. a–P200 effect, irony condition; b–P600 effect, irony condition; c–LFP, irony condition; d–LFP, mismatch condition.

Correlations between nonliteral response rates and effect amplitudes were run across all 106 participants in all three experiments. Fig 14 presents the results of these correlation tests.

The P200 (measured at Fz) showed no correlation, r = .03, *p* = .76. The P600 (measured at Pz) showed the strongest correlation, r = .28, *p* = .004. The LFP effect (measured at Fz) showed a small, near-significant correlation in irony condition, r = .18, *p* = .06, and a small, significant correlation in mismatch condition (nonliteral response rate calculated for mismatch condition for this correlation), r = .20, *p* = .04.

## General discussion

Table 1 shows a collapsed summary of which effects were statistically significant for the different conditions across the different experiments. The relevant effects are strongest over midline sites so the results summarized below are those from the midline ANOVAs.

**Table 1:** Summary of statistically significant effects across experiments.

	Condition	P200	P600	LFP
Experiment 1 Literal	Irony	*		*
	Mismatch			*
Experiment 1 Nonliteral	Irony	*	*	+
	Mismatch	*		*
Experiment 2	Irony	*	*	*
	Mismatch			*
Experiment 3	Irony	*	*	*
	Mismatch			*

*p < .05

^+^≤.06.

Results from three ERP experiments revealed that irony delivered by emojis elicits the same brain response as irony delivered by words, as reported in prior work. Robust P600 effects, as have been found in prior ERP studies of verbal irony, were consistently elicited by the wink emoji in our study in participants, and this effect was correlated with participants’ eventual interpretation of the sentences across all three experiments: participants who showed higher nonliteral (ironic) response rates to sentences followed by a wink emoji showed larger posterior P600 effects. The fact that most participants in Experiment 1 interpreted wink emoji sentences literally points to the previously-discussed ambiguity surrounding emoji use and interpretation and, additionally, the importance of broader context (e.g., background knowledge, speaker-listener common ground, etc.) in shaping the interpretation of both emojis and irony. Even though interpretations varied significantly across participants, those who did treat the wink emoji as a marker of irony reliably showed the P600 irony response. Previous offline studies have demonstrated that the wink emoji can be interpreted as a marker of irony; our neurocognitive evidence suggests that this irony is processed in the same way as verbal irony.

The P200 effects surfaced reliably to irony condition (the wink emoji) in every experiment. The P600 effect surfaced reliably to irony condition, but most strongly when the reader interpreted the sentence nonliterally. The LFP surfaced across experiments to both irony and mismatch conditions. The P200 and LFP effects were reliably frontal, the P600 reliably parietal.

Recent conceptions of the P600 have highlighted its general role, above and beyond morphosyntactic processing, in the processing of unexpected information from a range of domains (orthography, musical sequences, morphosyntax, event knowledge, etc.), and in reanalysis and reprocessing. This link with reprocessing has recently been highlighted by work employing co-registration of ERPs and eye movements during reading, which showed that P600s were specifically linked to regressive eye movements, signaling reanalysis [30]. Our present work shows important, novel extensions of this reprocessing account in two ways. First, the present study shows that language-related P600 effects can be elicited by non-verbal ideograms that convey meaning. This signals that the P600 is not uniquely associated with word-related anomalies in the language domain but can be elicited by linguistically-relevant ideograms requiring higher-level cognitive processing for integration. Findings from ERP studies of “visual language”–narratives told through comic strips–have found similar P600 effects to both anomalies in narrative constituent structure and incongruities that surface in new panels [47, 48]. Our work provides an important extension to related work on multi-modal communication, and in particular, links semantic and pragmatic aspects of emoji use with the same neural mechanisms used in higher-order language processing.

Second, correlation analyses showed that the presence and size of participants’ P600 effects is linked with the likelihood of nonliteral interpretations. This suggests that the P600 may specifically be tied to reanalysis of higher-level sentence meaning: when the literal meaning of a sentence is overridden in response to an ironic emoji, P600 effects ensue, such that the largest P600 effects are found in the participants who most frequently allowed nonliteral interpretations. This again mirrors findings from visual language studies, which found that more experienced comic strip readers demonstrated significantly larger P600 effects to the relevant anomalies [49]. These findings can be situated among other work on multimodal interactions and support theories including emojis and words as part of the same multimodal communicative system producing a single utterance as opposed to a sentence with an image added to it at the end [7]. This and other findings specifically on the P600 suggest this component is not specific to grammatical processing but rather a domain-general component reflecting reprocessing in structured, hierarchically organized systems (e.g., [47, 50, 51]).

A reliable P200 effect was found across all groups in all three experiments to the irony condition wink emoji, but generally not to the mismatch condition. This includes Experiment 3, where the mismatch (smile) and irony (wink) emojis were equally informative. This frontal P200 effect has been reported in previous ERP studies of verbal irony (though not always discussed), but the large P200 effect amplitude and the lack of correlation with behavioral responses in our studies complicates the interpretation of this finding. One possibility is that this component indexes early recognition of ironic meaning, which can possibly be followed by later re-processing of the representation of the sentence meaning (P600). Another possibility is that the wink emoji is simply more stimulating to the visual or attention systems than the smile and frown emojis. The first possibility is supported by the presence of the effect in other ERP verbal irony studies; the second is supported by the fact that there was no correlation between P200 effect amplitudes and behavioral response rate. That mismatch emojis generated no enhanced P200 effect, including in Experiment 3 where it was equally as informative as the wink emoji within the experimental context, could be consistent with either possibility–the lack of effect in this condition could be due to either the mismatch condition’s status as generally unconventional use of the smile and frown emojis, or the smile and frown emojis as less visually arousing. Note that these two explanations are not mutually exclusive, and these two potential causes may be working in combination. Further work can tease apart these two possibilities.

Emojis in the mismatch and irony conditions both showed enhanced late frontal positivities compared to expected match emojis. This ERP effect has been found in other language ERP studies in contexts where the time-locked word is plausible yet unexpected (e.g., “she pounded the nails with a *book*” [39], or specifically unpredicted word [38]; it has been hypothesized by some to reflect the processing cost associated with revising expectations and representations when specific lexical predictions are disconfirmed in the input [38]. While the wink emoji is conventionally used and recognized as a marker of irony, this is not the case with the smile or frown. The mismatch sentences and the emojis used therein have a variety of interpretations, such as communicating affect [52], that fall outside of the realm of irony processing. The fact that an enhanced LFP was found in both the mismatch and irony conditions consistently across all three experiments suggests that expectations for emojis may be qualitatively similar to those seen for predictable words, such that violations of specific expectations for both words and emojis elicit similar neural responses.

Our work is foundational in that it is the first to investigate the real-time neural correlates of emoji processing in sentence context, and link processing of irony elicited by words to irony elicited by sentence-embedded ideograms. The P600 effect elicited by ironic (wink) emojis is isomorphic to that seen in other studies of word-induced irony, suggesting a common set of processes involved in the comprehension and interpretation of irony, even in multimodal interactions. The late frontal positivity elicited by unexpected mismatching and ironic emojis also suggests that prediction mechanisms for the semantic or pragmatic content of emojis may be qualitative similar to those seen for words in informative contexts. Additionally, the very recent rapid rise of digital communications using ideographic enhancements means that conventional uses for these icons are likely still evolving and may vary between user communities. The present study, however, constitutes an important first piece of evidence of online emoji processing in utterance comprehension.
